# Introduction to ‘Making the most of AI’s potential: cross-disciplinary perspectives on the role of AI in science and society’

**DOI:** 10.1098/rsos.241306

**Published:** 2024-09-11

**Authors:** Marta Kwiatkowska, Mark Chaplain, Essi Viding

**Affiliations:** ^1^ Department of Computer Science, University of Oxford, Parks Road, Oxford OX1 3QD, UK; ^2^ School of Mathematics and Statistics, University of St Andrews, Mathematical Institute, St Andrews KY16 9SS, UK; ^3^ UCL, London, UK

We are pleased to introduce a special cross-disciplinary collection of manuscripts in *Royal Society Open Science* entitled ‘Making the most of AI’s potential: cross-disciplinary perspectives on the role of AI in science and society’. This collection of papers explores the use of machine learning, artificial intelligence (AI) and Big Data within science and society. These evolving fields will play an increasingly prominent part in not only the daily lives of individuals and societies, but also in how science is conducted, reported and applied. The papers bring together expert views on the applications and implications of these computational and statistical techniques. They also explore how science and societies will respond to these developments, alongside some of the ethical considerations the new technologies will bring to the fore.

We, the Editors of the Computer Science & Artificial Intelligence, Mathematics, and Psychology & Cognitive Neuroscience sections of *Royal Society Open Science,* commissioned this collection back in early 2020 to mark 10 years since the rise of the new era of AI. Unfortunately, the COVID-19 pandemic and subsequent lockdown caused an unprecedented delay, and the collection was temporarily halted. After 4 years, we are delighted to finally showcase the extraordinary depth and breadth of the research community within the field.

The papers initially published in this collection were invited by the Editors. However, no one could have predicted the tremendous developments in AI that occurred in the last couple of years and, to account for this, we would welcome future submissions. Subsequently, our long-term aim is to make this a ‘living collection’. We, therefore, anticipate seeing a constant stream of new papers added to the collection, as and when these are submitted to *Royal Society Open Science*. We have enjoyed reading and preparing these manuscripts and we are grateful that so many authors were willing to submit a paper to the collection; a special thank you to all the contributors.

Chawla *et al.* [1] reflect on the rise of AI across the course of the past 10 years. Taking a holistic perspective, they discuss the social and technical issues that have arisen as AI technology has been incorporated into our daily lives. Likewise, Lawrence and Montgomery [2] propose an engine for AI adoption in science. This is based on building supply chains of ideas across disciplines, advancing the capabilities of today’s AI systems, capability-building in interdisciplinary AI and a culture of open data science.

Gordon *et al.* [3] explore the safe use of robots, exoskeletons and other assistive technologies within society. They propose a framework for optimizing robot behaviour in triadic collaboration scenarios, which was found to significantly improve outcome measures for humans in robot-assisted tasks.

Several authors discuss the use of AI within health scenarios. Sanchez *et al.* [4] evaluate the advantages and challenges of using causal machine learning (CML) in healthcare and clinical settings. Similarly, Dang *et al.* [5] contribute a review into AI’s intersection with mobile health sensing, highlighting the role of key modalities such as audio, location and motion data.

The application of machine learning within modern physics was investigated by Alaa El-Din *et al.* [6], who combined existing physical models with machine learning, introducing a paradigm named surrogate training embedded in physics (STEP). This was discovered to show great potential for application within a range of modern physics processes.

It is evident that the increasing use of AI will have a major impact on the legal landscape. Koshiyama *et al.* [7] explore the AI regulatory environment, focusing on business reliance on algorithms. In response to a rising number of legal cases surrounding algorithms, they propose a new industry of Algorithm Auditing and Assurance to ensure the safe, ethical and legal use of AI within business. Likewise, Mittelstadt *et al.* [8] analyse the long-term legal risks of large language models (LLM). LLMs often generate mistruths, and the authors propose a pathway to create a legal duty for LLM providers to create models that are factually accurate and unbiased.

Jiang *et al.* [9] examine limitations in the capabilities of deep-learning models, particularly the limited quality of training data. The authors propose the issue of *generalized exploration* to conceptually combine exploration-driven learning between supervised learning and reinforcement learning.

Finally, Belle [10] discusses two formalisms that enable the integration of probabilistic programming and logic, and emphasizes their different strengths when tackling automated planning problems in complex domains.

We hope that readers of the journal will find papers in this collection of interest; we have certainly enjoyed editing the collection and have learnt much from those involved in this diverse and enthusiastic field. Taken together, we hope that the reader of this collection will not only gain a broader understanding of the wider scientific and societal implications of AI, machine learning and Big Data, but will also be inspired to conduct and submit their own related research. The papers curated in this collection are freely available at https://royalsocietypublishing.org/topic/special-collections/rsos-ai-cross-disciplinary.

Finally, we would like to thank the editorial office at *Royal Society Open Science* for all their help in preparing this collection.

## Data Availability

This article has no additional data.
